# Path analyses of cross-sectional and longitudinal data suggest that variability in natural communities of blood-associated parasites is derived from host characteristics and not interspecific interactions

**DOI:** 10.1186/s13071-015-1029-5

**Published:** 2015-08-19

**Authors:** Carmit Cohen, Monica Einav, Hadas Hawlena

**Affiliations:** Department of Life Sciences, Ben-Gurion University of the Negev, Beer-Sheva, 84105 Israel; Mitrani Department of Desert Ecology, Jacob Blaustein Institutes for Desert Research, Ben-Gurion University of the Negev, Midreshet Ben-Gurion, Israel

**Keywords:** Cross-sectional data, Fleas, Host age, Host reproduction status, Longitudinal data, Parasitic interspecific competition, Parasite community composition, Path analysis, Rodents, Vector-borne bacteria

## Abstract

**Background:**

The parasite composition of wild host individuals often impacts their behavior and physiology, and the transmission dynamics of pathogenic species thereby determines disease risk in natural communities. Yet, the determinants of parasite composition in natural communities are still obscure. In particular, three fundamental questions remain open: (1) what are the relative roles of host and environmental characteristics compared with direct interactions between parasites in determining the community composition of parasites? (2) do these determinants affect parasites belonging to the same guild and those belonging to different guilds in similar manners? and (3) can cross-sectional and longitudinal analyses work interchangeably in detecting community determinants? Our study was designed to answer these three questions in a natural community of rodents and their fleas, ticks, and two vector-borne bacteria.

**Methods:**

We sampled a natural population of *Gerbillus andersoni* rodents and their blood-associated parasites on two occasions. By combining path analysis and model selection approaches, we then explored multiple direct and indirect paths that connect (i) the environmental and host-related characteristics to the infection probability of a host by each of the four parasite species, and (ii) the infection probabilities of the four species by each other.

**Results:**

Our results suggest that the majority of paths shaping the blood-associated communities are indirect, mostly determined by host characteristics and not by interspecific interactions or environmental conditions. The exact effects of host characteristics on infection probability by a given parasite depend on its life history and on the method of sampling, in which the cross-sectional and longitudinal methods are complementary.

**Conclusions:**

Despite the awareness of the need of ecological investigations into natural host-vector-parasite communities in light of the emergence and re-emergence of vector-borne diseases, we lack sampling methods that are both practical and reliable. Here we illustrated how comprehensive patterns can be revealed from observational data by applying path analysis and model selection approaches and combining cross-sectional and longitudinal analyses. By employing this combined approach on blood-associated parasites, we were able to distinguish between direct and indirect effects and to predict the causal relationships between host-related characteristics and the parasite composition over time and space. We concluded that direct interactions within the community play only a minor role in determining community composition relative to host characteristics and the life history of the community members.

**Electronic supplementary material:**

The online version of this article (doi:10.1186/s13071-015-1029-5) contains supplementary material, which is available to authorized users.

## Background

The current view of community ecology is that host-parasite interactions should be studied in a realistic framework of multi-parasite and multi-host types reviewed by [1–3]. A large body of literature shows that environmental conditions and host characteristics, such as host species, body size, age, sex, reproductive state, genotype, and social rank, are important determinants of the community composition of parasites [4–18]. A different body of evidence highlights the importance of interspecific interactions among parasites in shaping parasite community composition reviewed by [19, 20]. For example, facilitation between micro- and macroparasites, resulting in richer parasite communities within vertebrate hosts [21], and competitive interactions between vertically transmitted pathogenic and non-pathogenic bacteria shape the community composition within arthropod vectors [20]. These interspecific interactions and their impact on community composition may be direct or may be mediated by the environmental conditions, by the host, or by a third party within the community [2, 22]. Regardless of the mechanism, interspecific interactions within parasite communities may influence the transmission and infection dynamics of pathogenic species and, consequently, impact infection risk in wildlife and in human populations (e.g., [23–31]). Accordingly, in order to reduce disease risk, researchers are currently developing techniques to manipulate the composition of natural communities of pathogens (e.g., [32–37]).

At present, environmental- and host-mediated effects are mostly studied separately from the effects of interspecific interactions (but see [24]). Moreover, most studies on interspecific interactions within a community of parasites have focused on a single guild (e.g., oral and gut bacteria, ectoparasites, or endoparasites; [22, 38–43]), whereas parasite communities in host individuals are naturally composed of multiple guilds. Finally, determinants of parasite community composition are mostly investigated via cross-sectional field surveys, through which parasites are sampled at one point in time from different host individuals and under different environmental conditions (e.g., [44–46]). Although cross-sectional field surveys allow a large sample size as they demand relatively low trapping efforts, they often fail to predict the actual relationships between variables [19]. Longitudinal sampling surveys, which repeatedly sample the same host individuals over time, are more reliable as they control for genetic variability between hosts and better reflect causality (i.e., the pattern in time at t+1 is a result of the pattern at time t; [19]). Importantly, longitudinal sampling always includes cross-sectional data at each time point. Yet, most longitudinal studies do not differentiate in their analyses between longitudinal and cross-sectional data (e.g., [47–50]). To the best of our knowledge, there is only one study that compared the ability of the two approaches in describing known relationships [19, 51]. However, since Fenton *et al.* were specifically interested in the interactions among parasite species, they did not quantify other determinants of parasite communities [19, 51]. Accordingly, three fundamental questions arise: (1) what are the relative roles of host and environmental characteristics compared with direct interactions between parasites in determining the community composition of parasites? (2) do these determinants affect parasites belonging to the same guild and those belonging to different guilds in similar manners? and (3) can cross-sectional and longitudinal analyses work interchangeably in detecting community determinants?

Here we addressed these three questions by sampling a natural rodent population, its fleas, ticks, and the two dominant vector-borne bacterial species on two occasions. The first occasion was the onset of the host reproductive period, when juveniles, reproductive and non-reproductive hosts occupied the same habitat, and the second was at the end of summer, when all juvenile individuals had reached adulthood and adults were no longer in a reproductive state. Thus, we examined both cross-sectional and longitudinal (based on two points in time) aspects of parasite community composition.

Blood-associated parasites share the same food resources (blood cells), may face similar constraints (e.g., immune response), and may rely on each other for transmission. Therefore, we hypothesized that interspecific interactions between parasites within such a community would be a major determinant of its composition ([52–54], but see, [55]). Moreover, while fleas and ticks constitute the ectomacroparasite guild (designated as the ectoparasite guild) and, as such, spend time both on the host and in the host environment, the bacteria constitute the endomicroparasitic guild, i.e., located and reproducing within a host/vector individual, and are thus highly associated with it (designated as the endoparasite guild). Consequently, we predicted that whilst all the sampled blood-associated parasites would be affected by the host characteristics, fleas and ticks would be more influenced by the external environment and would show more seasonal fluctuations. Finally, as the cross-sectional and longitudinal approaches have different strengths and weaknesses [56], we hypothesized that they would be complementary in evaluating the determinants of parasite community composition.

By combining path analysis and model selection approaches, we explored the multiple direct and indirect paths that connect (i) the environmental and host-related characteristics to the infection/infestation probability of a host by the four parasite species, and (ii) the infection/infestation probabilities of the four species by each other. Our results suggest that the majority of paths shaping the community composition of parasites are indirect, mostly determined by host characteristics and not by interspecific interactions or environmental conditions. The exact effects of host characteristics on infection probability by a given parasite depend on its life history and on the method of sampling, in which the cross-sectional and longitudinal methods are complementary. Thus, our study supports the central role of the immediate environment of a parasite community in shaping its composition and calls for controlled exploration of the mechanisms underlying host-related effects.

## Methods

### Experimental design

We trapped, tagged and identified *Gerbillus andersoni* and *Gerbillus pyramidum* rodents and sampled their fleas, ticks and blood on two occasions, namely at the onset of the host reproductive period (April-May; period 1) and at the end of summer (September; period 2), when all juvenile individuals had reached adulthood. All blood samples were subjected to DNA extraction and polymerase chain reaction (PCR) to screen for the most dominant vector-borne bacteria within the rodent blood. We then combined path analysis and model selection approaches [57, 58] to determine the most important direct and indirect determinants of the community composition of the parasites (ticks, fleas, and two bacterial species). This protocol allowed us to evaluate the relative importance of determinants related to the host, to interactions among parasites within the host, and to the host environment. It also allowed for the simultaneous evaluation of the determinants of parasite community composition by cross-sectional and longitudinal analyses. The longitudinal analysis was based on the comparison of parasite communities sampled during the two sampling periods in the same rodent individuals. To get a comparable sample size while maximizing the heterogeneity in host and environmental conditions, the cross-sectional analysis was based on the comparison of parasite communities sampled during period 1 in hosts belonging to different age and sex groups and that differed in their reproductive status.

### Rodent trapping and sampling

To make the study more general and not restricted to a single host population, we aimed at sampling at least 10 independent rodent populations. Plots were located in the western Negev Desert, Israel (Hevel Shalom, 31^0^ 10′ N) and included 40 live Sherman traps, uniformly placed in four columns and 10 rows per plot. We maintained plot independence by selecting the 10 plots to be at least 40 m apart from each other, following Kedem *et al.* [59]. At the same time, for the longitudinal portion of the study, we aimed at 80 % recaptures per plot, which required two to three trapping nights within each plot.

We recorded temperatures in the evening and morning of trapping using a Kestrel 4000, Pocket Weather Tracker (Nielsen-Kellerman, Boothwyn, Pennsylvania, USA) and calculated mean values in each plot. We tagged each captured individual and determined its species, sex, age (juveniles <18 g; adults >18 g, following Hawlena *et al.* [60]) and, in female rodents, the reproductive status (pregnant, lactating or non-reproductive hosts). We then weighed each gerbil and measured the length of its right hind leg, and we used the two measurements for the calculation of body condition, following J Peig and AJ Green [61]. Within each of the two sampling periods, we collected fleas from rodent individuals only following their initial capture to avoid pseudo-replication. The rodent was held gently above a plastic can, its fur was blown, and jump-off fleas were counted and collected, until no fleas were detected on the host body. Ticks were often attached to ears, mouth or nose and were aggregated around open injuries. This aggregation pattern allowed us to count larvae and nymph ticks on a gerbil body by carefully scanning the target areas before collecting them, using sharp-tip forceps. Despite our efforts to screen for all ectoparasite species that could act as vectors for bacteria, we never detected lice on the rodents in our study areas. Fleas and ticks were stored in 70 % ethanol at −20 °C until their species and stage (for ticks) were identified. In addition, we drew 100–200 μl of blood from the retro-orbital sinus of each host individual with capillaries immersed in 0.15 % EDTA and stored each sample in EDTA blood collection tubes at −20 °C until DNA extraction. Specifically, the rodent was held gently on a covered foam pillow, one drop of local anesthesia (Localin, 0.4 % benoxinate hydrochloride; Fisher Pharmaceutical Labs, Tel Aviv, Israel) was administered to the eye, and shortly after, the capillaries were introduced into the retro-orbital sinus. As some species of wild rodents are extremely sensitive to slight deviations in anesthetic doses [62], this protocol ensured that the rodents would have no comprehensive deficits when released back into nature while minimizing discomfort to the animals.

The trapping and handling protocol was approved by the Committee for the Ethical Care and Use of Animals in Experiments of Ben-Gurion University of the Negev (permission # IL-14-03-2011) and by the Nature and National Parks Protection Authority (permission # 2011/38146).

### Bacteria sampling

We extracted DNA from blood samples, using a MoBio Bacteremia DNA Isolation Kit. We added 50 μl of blood to the microbead tube and followed the manufacturer’s instructions. We chose *Mycoplasma* and *Bartonella* as the target genera for the study because the 16S rRNA pyrosequencing of the DNA extracted from gerbil blood and fleas suggests that they dominate the blood-borne bacterial communities [63, 64]. To confirm the existence of *Mycoplasma* and *Bartonella* in the vector arthropods, we also extracted DNA from 126 fleas and 42 ticks, using a DNeasy Blood and Tissue Kit (QIAGEN, Valencia, CA, USA), according to the manufacturer’s instructions for the purification of total DNA from ticks, and the supplementary protocol for the detection of *Borrelia* DNA [65]. In each extraction session, a negative control was added in which all of the reagents were added to phosphate-buffered saline instead of blood or vectors.

The phylogenetic tree of the specific *Bartonella* species detected in these areas is described elsewhere and suggests that it comprises multiple genotypes [59, 66]. In contrast, a phylogenetic analysis suggests that the *Mycoplasma* bacteria belong to a single cluster, with a 90–95 % similarity to *Mycoplasma haemomuris* [59]. Detection of bacterial species in DNA extracts relied on PCR. We detected *Mycoplasma* by amplification of the 16S gene with HM16S-1(fw) (GAGCGAATTGCTAGCAATAG) and HM16S-2(rev) (AGCTACAACGCTGAGACTC) primers. We detected *Bartonella* by amplification of the citrate synthase (*gltA*) gene withBhcs.781p-fw (GGGGACCAGCTCATGGTGG) and Bhcs.1137n-rev (AATGCAAAAAGAACAGTAAACA). The PCR conditions are described in Kedem et al., [59]. Despite the uniform DNA extraction and PCR protocol, the intensities of the positive PCR bands of *Bartonella* were variable, ranging from very weak bands that could hardly be seen to definitive bands. We, therefore, exploited this variability to distinguish between samples with low (<500 copies per 1 μl of DNA) and high (>500 copies per 1 μl of DNA) *Bartonella* sp. cell density, with this distinction serving as the binary response variable in subsequent analysis. To distinguish between low and high cell density, we ran, in parallel to the PCR assays, an intensity ladder of *Bartonella* (ranging from 10 to 5000 bacterial copies in 1 μl of DNA) that was made by a serial dilution of a positive control with a known copy number.

Sanger sequencing was performed on 20 % of the PCR-positive samples, using a PRISM 3100 Genetic Analyzer (Applied Biosystems, Carlsbad, CA, USA) at the National Institute for Biotechnology in the Negev, Beer-Sheva, Israel and confirmed that the tested bands indeed corresponded to sequences derived from *Mycoplasma haemomuris*-like bacterium and *Bartonella* sp.

### Data analysis

**Fig. 1:**
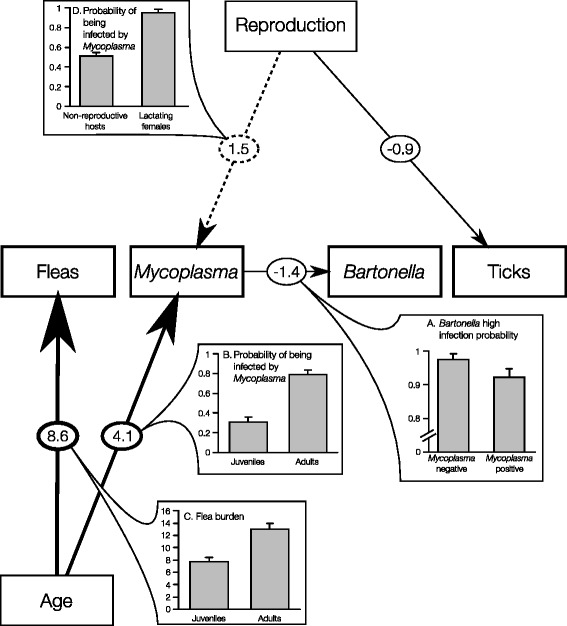
The best path analysis model for predicting the community composition of parasites as determined by the cross-sectional dataset (model 12 in Additional file 1: Table S1). *Arrows* represent direct and indirect influences. Numbers on the arrows are standardized path coefficients, representing the relative strength of the given effect (β/SE), which is also reflected by the arrow width. **a–d** illustrate the directions of the most influential (|β/SE| > 1) direct effects. The *dashed arrow* represents a relationship that is included in the second best model in addition to the relationships included in model 12 (model 14 in Additional file 1: Table S1)

**Fig. 2:**
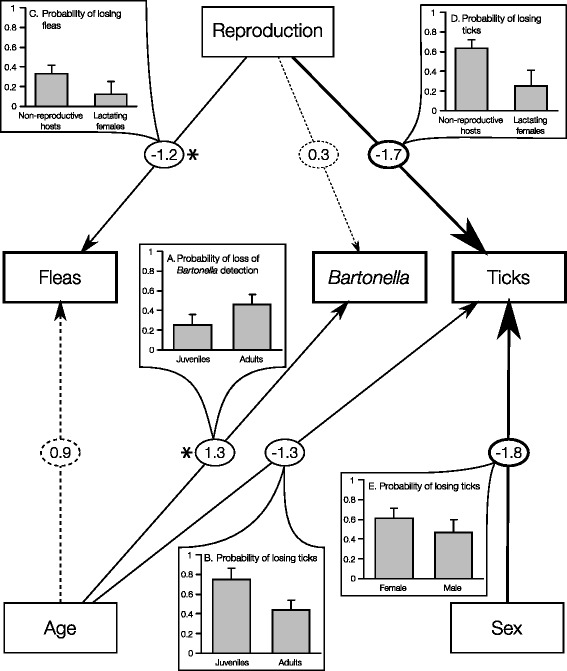
The best path analysis model for predicting the temporal changes in the community composition of parasites as determined by the longitudinal dataset (model 48 in Additional file 1: Table S1). Arrows represent direct and indirect influences. Numbers on the arrows are standardized path coefficients, representing the relative strength of the given effect (β/SE), which is also reflected by the *arrow* width. **a–e** illustrate the directions of the most influential (|β/SE| > 1) direct effects. The *dashed arrows* represent additional relationships that are included only in the second and third best models (models 49 and 46, respectively, in Additional file 1: Table S1). Asterisks denote relationships that were not included in the second and third best models

Analyses for each of the datasets were conducted in two stages: first, we searched for the most important factors that may have influenced each parasite species (Table 1); then we quantified the causal pathways in the network of interactions among the four parasites and their determinants, using a combination of path analysis and model selection approach (Additional file 1: Table S1, Figs. 1, 2 and Additional file 1: Figure S1). Path analysis is a powerful approach that evaluates alternative causal hypotheses regarding the interactions among variables. The causal links that this analysis reveals are often supported by an experimental approach (e.g., [58]). The model selection approach complements the path analysis by evaluating the likelihood of the causal hypotheses, which reflect different predictions about the directions and strength of interactions, given the data and the set of models. To this end, in addition to the insights gained from traditional correlation-based approaches about the strength and significance of pairwise interactions, the combined path analysis-model selection approach can incorporate multiple interrelated response variables, can predict the direction and causality of the interactions, and can distinguish between direct and indirect effects. Accordingly, the combined approach enabled us to make comparisons between models with and without direct interactions between parasites (question 1), to quantify and compare the relative importance of host characteristics on each of the parasite species in the community (question 2), and to qualitatively compare the cross-sectional and the longitudinal datasets (question 3). In both stages, we compared models using model probabilities (w_i_, where i corresponds to a specific model) based on Akaike’s information criterion corrected for a small sample size (AICc), which gives a measure of the plausibility, on a 0 to 1 scale, that a particular model is indeed the best model see model selection approach; [57]. The competing models were based on generalized linear models (GLM) with a binary-binomial distribution for the binary-response variables and a negative binomial distribution for the count-response variables.

**Table 1:** Comparison of models from stage 1

Response variables included in the model		Dataset and target parasite							
Type	Variable	Cross-sectional				Longitudinal			
		M	B	S	H	M	B	S	H
Environment	^a^Temperature	0	0	0.02	0	n/a	n/a	0	0
Host	^b^Age	**17**	**27**	**96**	0.2	n/a	**25**	**28**	**32**
	^b^Body condition	0	0	0	0	n/a	0	0	0
	^b^Reproductive status	**35**	**22**	0	**99**	n/a	**29**	**43**	**43**
	Sex	**13**	**24**	0	0	n/a	**23**	**29**	**29**
Vector	^b^Flea burden	0	0	n/a	0	n/a	0	n/a	0
	^b^Tick burden	0	0	0	n/a	n/a	0	0	n/a
Bacteria	^b^ *Mycoplasma* presence	n/a	**27**	n/a	n/a	n/a	**23**	n/a	n/a
	^b^ *Bartonella* presence	**35**	n/a	n/a	n/a	n/a	n/a	n/a	n/a

The models explain occurrence/abundance or temporal reduction for each of the four parasitic species in the cross-sectional and longitudinal datasets, respectively. Values are weights (*w*_*i*_) in percentages of Akaike information criterion corrected for sample size—the relative likelihood of the current model, given the data and the set of models. Weights are normalized across the set of candidate models to summate to one, and are interpreted as probabilities. M = *Mycoplasma haemomuris*-like bacterium, B = *Bartonella* sp., S = *Synosternus cleopatrae* fleas, H = *Hyalomma impeltatum* ticks, n/a = not applicable. The best models (*w*_*i*_ > 10) are marked in bold and were used for stage 2 (Additional file 1: Table S1, Figure S1)

^a^For the longitudinal analyses, we used the between-period temperature differences

^b^For both datasets, we used the relevant factor measured in the first period

The independent variables considered in stage 1 were similar for both datasets and included environmental, host-related, vector-related, and bacteria-related variables (Table 1). For the cross-sectional analyses, we used either “the prevalence of infection by the endoparasites” or “the abundance of ectoparasites” as response variables, whereas for the longitudinal analyses, we used binary-response variables, indicating whether there was a reduction in infection/infestation between periods or not (i.e., infection/infestation either stayed stable or decreased).

Stage 2 began with the construction of saturated path models [58, 67] that integrate all important associations between the independent variables and each single parasite (i.e., all associations included in the best models from stage 1; Additional file 1: Figure S1). For each dataset, we then compared different model versions modified from the saturated models while keeping the same AIC metric (i.e., using the same mediators and response variables) (Additional file 1: Table S1 and Figure S1). Path analyses were conducted using the Mplus software (Muthén & Muthén, Los Angeles, CA, USA).

We performed goodness of fit chi-square tests to assess whether the infection/infestation status (occurrence or abundance) of each parasite species is likely to change with time. We compared the observed and the expected changes. The expected changes were calculated based on the random probabilities that an infection/infestation is either (1) amplified ((1-p_i_)× p_i_, where p_i_ is the probability for a host to be infected/infested by parasite i during the period 1), (2) reduced (p_i_× (1-p_i_)) or (3) not changed (p_i_^2^+(1-p_i_)^2^) with time.

## Results

### General description of the rodent population and its parasitic community

Of a total of 511 individual rodents captured in all 10 plots, *G. andersoni* constituted 97 % (*N* = 496) and *G. pyramidum* constituted 3 % (*N* = 15); we thereby focused our analyses on the former species. Details on the *G. andersoni* gerbils who were trapped in period 1 (*N* = 339) and the blood samples included in the cross-sectional and longitudinal datasets are given in Table 2. As expected, most juvenile and lactating females were trapped during the spring (period 1), and by the end of the summer (period 2), the rodent population had homogenized (with only 14 new juveniles and no reproductive adults).

**Table 2:** Summary of field sampling

	Host characteristics			Total rodents	Infection status				Samples for DNA extraction		
Dataset	Age	Sex	Reproductive status		S intensity (prevalence)	H intensity (prevalence)	M Prevalence	^a^B Prevalence	Blood samples	Flea samples	Tick samples
Cross-sectional	Juveniles	Male	NR	64	8 (98)	6 (50)	36	95	41	14	5
		Female	NR	67	7 (100)	9 (57)	24	100	37	16	10
	Adults	Male	NR	84	13(97)	8 (42)	77	97	40	16	7
		Female	NR	59	16(100)	7 (54)	65	92	37	16	11
		Female	R	65	11 (97)	9 (17)	95	87	40	0	4
Longitudinal	Juveniles	Male	NR	9	15(100)	5(55)	57	86	14	30	2
		Female	NR	13	10(100)	6(58)	33	88	18	32	3
	Adults	Male	NR	28	15(100)	7(45)	87	75	16	32	5
		Female	NR	19	15(100)	5(50)	44	78	18	32	3
			R	9	15(100)	7(28)	87	62	16	0	0
	**Total**			**417** ^**b**^	**125 (99)**	**69** ^**e**^ **(46)**	**61**	**86**	**277** ^**c**^	**188** ^**d**^	**50** ^**c**^

Details on the trapped *G. andersoni* rodents, on the prevalence (percentage of infected/infested hosts) and intensity (mean abundance of ectoparasites per infested host) of their *Mycoplasma* (M), *Bartonella* (B), *S. cleopatrae* (S), and *H. impeltatum* (H) parasites, and on the samples included in the cross-sectional and the longitudinal datasets

^a^99% of the hosts were infected by *Bartonella;* thus we exploited the variability in the intensities of positive PCR bands to distinguish between samples with low (<500 copies per 1 μl of DNA) and high (>500 copies per 1 μl of DNA) *Bartonella* sp. cell density. This distinction served for the prevalence calculation that in the case of *Bartonella,* corresponded to the percentage of highly infected hosts

^b^We had in total 339 *G. andersoni* captured in the first period, but 78 of them are relevant to both datasets

^c^We had in total only 236 blood samples, but 41 of them are relevant to both datasets

^d^We had in total 126 flea samples, but 62 of them are relevant to both datasets

^e^We had in total 42 tick samples, but 8 of them are relevant to both datasets

During period 1, most fleas were morphologically identified as *Synosternus cleopatrae*. There was also a low prevalence of the winter flea, *Stenoponia tripectinata* (none in juveniles and 9 % in adults) at this time, leading to the inclusion in the longitudinal analysis of only host individuals who were not infested by the winter flea. During period 2, all host individuals were solely infested by *S. cleopatrae*. All larvae and nymph ticks were morphologically identified as *Hyalomma impeltatum.* The majority of hosts were infected/infested by more than one parasitic species (23, 55, and 19 % were infected/infested by two, three, and four parasites, respectively). Almost all the rodent individuals were infested by fleas and were highly infected by *Bartonella*, whereas the prevalence of ticks and of *Mycoplasma* was lower (Table 2). *Bartonella* and *Mycoplasma* were also detected in fleas (65 and 33 %, respectively) and in the tick samples (38 and 19 %, respectively), implying that these bacteria have the potential for vector-borne transmission.

The temporal fluctuations of the different parasite species on a given host individual were dissimilar. *Mycoplasma* status was mostly persistent, with 90 % of the individual rodents remaining at the same status of infection (uninfected, *n* = 15 or infected, *n* = 22) between the two periods. In fact, only one individual acquired a new infection, and in three individuals that were positive in period 1, no *Mycoplasma* was later detected in the peripheral blood. Due to this persistency, we did not include *Mycoplasma* in the longitudinal analyses. Similarly, most (58 %, *n* = 24) rodents were infected by *Bartonella* in both sampling seasons. However, 38 % (*n* = 15) were positive in period 1 but showed no evidence of *Bartonella* infection in the peripheral blood in period 2. For the other two individuals that were sampled, one remained PCR negative and the other became PCR positive in period 2. Regarding ectoparasites, most host individuals had either a higher flea burden (68 %, *n* = 27) or a lower tick burden (58 %, *n* = 23) in period 2 than in period 1. The numbers of hosts with either a persistent *Mycoplasma* infection, an increased flea burden, a reduced tick burden, or a loss of detection of *Bartonella* in the PCR of peripheral blood (hereafter, “loss of detection”) were greater than expected by a random chance (chi-square tests: *χ*^2^ > 22, *p* < 0.001, for all parasites).

### Determinants of parasite community composition

The best models predicting the infection/infestation status of a rodent by each parasite alone and the chances that it will change over time are detailed in Table 1. The best models predicting the prevalence of *Mycoplasma* and *Bartonella* included the effect of co-infection with the other bacterium. However, other than this, all the best models predicting the infection/infestation status of a rodent by any of the parasites included only host characteristics. Models including other direct interactions between parasites (e.g., between fleas and ticks or between both of these and the bacterial species) or temperature effects performed poorly compared to models including host characteristics (Table 1), and thus were not included in any of the path models (Additional file 1: Table S1).

When we identified the best path models, some of the variables included in the best single-species models were omitted (Table 1 versus Figs. 1 and 2). For example, the effect of the host sex was not included in the best path model of the cross-sectional dataset and remained directly linked only to the probability of losing ticks in the path model of the longitudinal dataset. Moreover, the path analysis for the cross-sectional dataset indicates that the observed associations between host characteristics (age, sex and reproductive status) and *Bartonella* prevalence (Table 1) probably arose due to direct relationships between the two bacterial species rather than by direct effects of the host characteristics on *Bartonella* (Fig. 1a).

From all the path models considered for the cross-sectional dataset (Additional file 1: Table S1 and Figure S1), only two—*Mycoplasma*-centered—models had a good support of the data (∑*w*_*i*_ = 100; Fig. 1). These models suggest that mainly four direct relationships determine the community composition of parasites (the absolute relative strength of effects are larger than 1; Fig. 1). These relationships include the negative relationships between the two bacterial species (Fig. 1a), and the positive relationships between (i) *Mycoplasma* prevalence and host age (Fig. 1b), between (ii) flea burden and host age (Fig. 1c), and between (iii) *Mycoplasma* prevalence and the reproductive status of the host (Fig. 1d). From all the path models considered for the longitudinal dataset (Additional file 1: Table S1, Figure S1), three models had a good support of the data (models 46, 48–49 in Additional file 1: Table S1; ∑*w*_*i*_ = 78; Fig. 2). These models suggest that mainly five direct relationships determine the temporal changes in the community composition of parasites (the absolute relative strength of effects are larger than 1; Fig. 2). These relationships include positive relationships between *Bartonella* loss of detection and host age (Fig. 2a) and negative relationships between (i) host age and tick loss (Fig. 2b), (ii) between reproductive status and flea loss (Fig. 2c), between (iii) reproductive status and tick loss (Fig. 2d), and between (iv) host sex and tick loss (Fig. 2e).

## Discussion

The assembly of blood-associated parasites in a host individual presents a highly interrelated community, which may be shaped by both extrinsic (environmental and host characteristics) and intrinsic (interspecific interactions) factors. Here we quantified the relative impacts of environmental, host-related and vector-related factors and compared them to the relative impact of interspecific interactions on the composition of the most dominant parasites forming these communities. Importantly, we assessed the contribution of cross-sectional and longitudinal approaches to the investigation of the determinants of community composition. We discuss below our results in light of the three study goals.

### The community composition of blood-associated parasites is mostly determined by rodent characteristics and not by interspecific interactions among parasites

Reports on interspecific interactions within ecto- or endoparasite communities are common (e.g., [24, 26, 43, 68–73]), but they are mostly based on traditional correlative studies [19, 74]. Here we employed a path analysis approach to determine causality from correlative data [58]. Although the path analysis cannot replace experimental evidence in determining causation, this method, combined with the model selection approach, has a predictive power for the direction, strength, and nature (direct versus indirect) of the relationships among variables. As expected, we found indications for direct negative associations between the two bacterial species (Fig. 1a). Given that *Mycoplasma* bacteria reside at the erythrocyte surface [75], it is possible that the two bacterial species compete on attachment sites during the process of erythrocyte invasion by *Bartonella*.

Nevertheless, in contrast to our hypothesis, our analyses suggest that all other associations within the tested parasite community are indirect and are mediated by host characteristics. While these results fit well with the known nature of intimal host-parasite associations and with the important role of indirect effects in shaping complex natural communities [76, 77], they challenge the prevailing claim, based on field correlative studies, that mainly interspecific interactions among parasites shape their community composition, and they thus call for experimental evaluations of the field patterns. Specifically, our cross-sectional analysis indicates that the associations between *Mycoplasma* and fleas or ticks are mediated by host characteristics (Fig. 1). The failure to detect direct positive associations between *Mycoplasma* and the arthropod vectors, combined with unsuccessful flea-transmission experiments in different *Mycoplasma* species (Cohen, C.*et al.*, and Lappin M.R. unpublished observations) and evidence for alternative routes of transmission e.g., via aggressive host interactions and *in utero* transmission; [78–81], suggests that arthropods may play only a secondary role in haemoplasma transmissions. Similarly, our results suggest that the associations between the two ectoparasites are mediated by host characteristics (Fig. 2). Negative indirect associations were also revealed by Hawlena *et al.* [69] who suggested that ticks may modify the flea preference for specific host types.

However, in contrast to the above findings, which reflect the current knowledge on *Mycoplasma*-vector and flea-tick interactions, the lack of evidence for direct relationships between *Bartonella* and fleas is most puzzling. This is because fleas are considered as key players in the *Bartonella* cycle based on their dominancy in flea microbiomes [64, 65, 82] and conclusive experimental evidence of efficient flea-borne transmission (e.g., [83–85]). In some other observational studies, the correlations between flea abundance and *Bartonella* occurrence were weak or also absent [49, 66, 86]. The reason that direct positive effects between *Bartonella* and fleas may be obscured in observational data could be attributed to the overall high prevalence of *Bartonella* and/or direct interaction of *Bartonella* with other bacteria that are less dependent on fleas for their transmission (*Mycoplasma* in our case).

### Determinants for the occurrence/abundance of a parasite depend on its life history and not on its guild

Our hypothesis that ectoparasites would be largely affected by the external environment of the host, and thus would show more seasonal fluctuations than endoparasites was only partly supported. As expected, the abundances of both ectoparasite species varied seasonally, but contrary to our hypothesis, they were mostly predicted by the host characteristics. Moreover, none of the best models included temperature, suggesting that the seasonal patterns in ectoparasite abundance were probably derived from changes in host characteristics (Table 1).

Despite the fact that all four parasite species were mostly affected by the host age and reproductive status, major differences were revealed among them with respect to the relative impact of each host characteristic, the direction of effects (i.e., positive versus negative), and the seasonal patterns. *Mycoplasma* mostly infected adults, in particular, lactating females, and were persistent in a host through time. The observed persistency is consistent with the dynamics of this bacterial species in the 30 field-captured *G. andersoni* that we have introduced to our flea-free lab and have maintained as continuously infected over 3 years (Cohen *et al.*, unpublished observations). The long persistency and multiple routes of transmission reviewed in [87] can explain why adults who (i) live longer, (ii) support more fleas (Fig. 1c), and (iii) are more likely to be engaged in aggressive mating (including reproductive females) have a higher *Mycoplasma* prevalence than do juvenile rodents.

In contrast, our data suggest that *Bartonella* prevalence is not directly affected by any of the host characteristics. Moreover, 38 % of the rodents were positive for *Bartonella* in the first period but became PCR-negative after only 3–4.5 months. The duration of *Bartonella* bacteremia in nature is difficult to determine due to relapsing bacteremia from the primary niche and reinfections promoted by fleas [88]. Yet, our results are consistent with controlled inoculations under flea-free conditions, which suggests that after a period ranging from several weeks to several months, *Bartonella* infection is cleared by the host or is at least reduced to undetectable quantities [88–91]. It is still not clear whether the clearance mechanisms of *Bartonella* bacteria involve cellular immunity (e.g., T helper cells, B cells, or cytokines), humoral immunity (specific antibodies in serum that can remove *Bartonella* from the bloodstream), an acceleration of erythrocyte turnover, or their combination [88]. However, it is likely that these mechanisms are age-dependent (Fig. 2a; reviewed by [92]).

Interspecific differences were also revealed between the two ectoparasites. Flea burden was mostly predicted by the host age, with higher rates in adults than in juvenile hosts. This result, which is consistent with previous field patterns and with experimental evidence for a higher grooming efficiency in juveniles than in adults [60, 93], may explain the increase in flea burden toward period 2. In contrast, tick burden was mostly affected by the reproductive status of the host. The results, which indicate that lactating females are more resistant to ticks than non-reproductive adults, apparently contradict the collective evidence for a “cost of reproduction” (i.e., lower immunity in reproductive than in non-reproductive females; [94]). However, the lower probability of lactating females to lose ticks in the following season, compared with non-reproductive females, suggests that the cost of reproduction may be simply postponed.

Taken together, our results suggest that while all blood-associated parasites are mainly affected by host characteristics, the relative roles of different host characteristics vary with respect to their life history.

### Cross-sectional and longitudinal sampling are complementary approaches

As predicted, we found that the two approaches are complementary and together give a comprehensive view of the determinants of parasite community composition. From a quantitative perspective, the cross-sectional analysis revealed fewer but stronger (i.e., higher effect sizes) paths between host characteristics and parasite occurrence/abundance than did the longitudinal analysis. The stronger effects of the cross-sectional analysis may reflect differences in time scales, since via the cross-sectional analysis, we compared parasite communities in hosts composed of two different age cohorts (i.e., newly born juveniles versus adults from the previous year), and via the longitudinal analysis, we compared the parasite communities of hosts before and a few months after they became mature. The higher sensitivity of the longitudinal analysis may be attributed to the removal of between-individual noise [56]. From a qualitative perspective, utilizing the two approaches allowed us to reveal possible paths and effects that would otherwise have been missed. The age effects in fleas and interspecific associations between bacteria were uncovered by the cross-sectional analysis, while the longitudinal analysis provided evidence solely for (i) the cost of reproduction suggested by a lower resistance to ticks, (ii) the age and sex effects in ticks, and (iii) the loss of detection of *Bartonella* by adult hosts. Our longitudinal survey was limited to two time points, but this sampling is reasonable given the one-year lifespan of *G. andersoni* under natural conditions [95]. Importantly, we managed to capture the main variability in physiological age and reproductive status by sampling rodents in both the reproductive and the non-reproductive seasons. Thus, we believe that the combination of cross-sectional and longitudinal approaches generated a comprehensive view of blood-associated parasite communities of rodents in nature.

## Conclusions

In the past few decades, the role of ecological investigations of natural host-parasite communities has gained remarkable attention in light of the emergence and re-emergence of infectious diseases [96–99]. Vector-borne zoonotic diseases, in particular, present a challenge due to the complexity of the paths that their agents use and the great variety of factors that can directly and indirectly affect their occurrence [25]. The network of effects and mechanisms would be best revealed by lab and field manipulations. However, in practice, such studies are often not practical (e.g., for dangerous zoonotic diseases and long-term scales). Here we illustrated that comprehensive patterns can be revealed from observational data by applying path analysis and model selection approaches and combining cross-sectional and longitudinal analyses. By employing this combined approach on blood-associated parasites, we concluded that direct interactions within the community may play only a minor role in determining community composition relative to host characteristics and the life history of the community members. Our combined approach is expected to generate important insights into the structure and function of other host-vector-parasite systems, in particular, and into natural communities with complex causal relationships, in general.

## Additional file

Additional file 1: Table S1. List of path models and their weights (in percentages) employed in stage 2. **Figure S1**. Basic path models used for the construction of the set of competing models (Additional file 1: Table S1). (DOCX 128 kb)
