# Supplementary material for: Path analyses of cross-sectional and longitudinal data suggest that variability in natural communities of blood-associated parasites is derived from host characteristics and not interspecific interactions
Source: Parasit Vectors. 2015 Aug 19;8:429. doi: 10.1186/s13071-015-1029-5 (PMC4545369; doi:10.1186/s13071-015-1029-5)
Supplement: Additional file 1: Table S1. — List of path models and their weights (in percentages) employed in stage 2. Figure S1. Basic path models used for the construction of the set of competing models (Additional file 1: Table S1). (DOCX 128 kb) [file 13071_2015_1029_MOESM1_ESM.docx]

Supplementary material

**Table S1**. **List of path models and their weights (in percentages) employed in stage 2**

The models were set to predict the community composition of parasites associated with *G. andersoni* rodents and its seasonal changes. Weights are for Akaike information criterion corrected for sample size (AICc). Models include the saturated models and variations of their derivatives (i.e., *Mycoplasma*-centered, *Bartonella*-centered, and indirect interaction models; Fig. S1). The common feature for all models is that they include the most important associations revealed in stage 1 (marked by bold in Table 1). All models constructed for a given dataset are based on the same AIC metric (i.e., using the same mediators and dependent variables) and therefore are comparable. *w_i_* = Akaike weights—the relative likelihood of the current model, given the data and the set of models—which are normalized across the set of candidate models to summate to one, and are interpreted as probabilities. The best models (*w_i_* > 10%) are marked in bold and are presented in Figs.1 and 2.

| Dataset | Model basis | # | Model details | *w_i_*^1^ |
| --- | --- | --- | --- | --- |
| Cross-sectional | Saturated model | 1 | Fig. S1A | 0 |
|  |  | 2 | Fig. S1A with *Bartonella* effect on *Mycoplasma* instead of vice versa | 0 |
|  |  | 3 | Fig. S1A, excluding the effect of sex on *Mycoplasma* | 0 |
|  |  | 4 | Fig. S1A, excluding the effect of sex on *Bartonella* | 0 |
|  |  | 5 | Fig. S1A, excluding the effect of sex on *Mycoplasma* and *Bartonella* | 0 |
|  |  | 6 | Fig. S1A, excluding the effects of age on *Mycoplasma* and sex on *Bartonella* | 0 |
|  |  | 7 | Fig. S1A, excluding the effects of reproductive status on *Mycoplasma* and sex on *Bartonella* | 0 |
|  |  | 8 | Fig. S1A, excluding the effects of age and sex on *Bartonella* | 0 |
|  |  | 9 | Fig. S1A, excluding the effects of reproductive status and sex on *Bartonella* | 0 |
|  |  | 10 | Fig. S1A, with *Bartonella* effect on *Mycoplasma* instead of vice versa, excluding the effects of age and sex on *Bartonella* | 0 |
|  | *Mycoplasma*-centered | 11 | Fig. S1B | 0 |
|  |  | **12** | **Fig. 1, excluding dashed arrow** | **54** |
|  |  | 13 | Fig. S1B, excluding the effects of sex and age on *Mycoplasma* | 0 |
|  |  | **14** | **Fig. 1, including dashed arrow** | **46** |
|  | *Bartonella*-centered | 15 | Fig. S1C | 0 |
|  |  | 16 | Fig. S1C, excluding the effect of sex on *Bartonella* | 0 |
|  |  | 17 | Fig. S1C, excluding the effects of sex and age on *Bartonella* | 0 |
|  |  | 18 | Fig. S1C, excluding the effects of sex and reproductive status on *Bartonella* | 0 |
|  | Indirect interactions | 19 | Fig. S1D | 0 |
|  |  | 20 | Fig. S1D, excluding the effect of sex on *Mycoplasma* | 0 |
|  |  | 21 | Fig. S1D, excluding the effect of sex on *Bartonella* | 0 |
|  |  | 22 | Fig. S1D, excluding the effect of sex on *Mycoplasma* and *Bartonella* | 0 |
|  |  | 23 | Fig. S1D, excluding the effects of sex and reproductive status on *Mycoplasma* and *Bartonella* | 0 |
|  |  | 24 | Fig. S1D, excluding the effects of age and reproductive status on *Mycoplasma* and *Bartonella* | 0 |
|  |  | 25 | Fig. S1D, excluding the effects of age and sex on *Mycoplasma* and *Bartonella* | 0 |
|  |  | 26 | Fig. S1D, excluding the effect of sex on *Mycoplasma* and the effects of sex and age on *Bartonella* | 0 |
|  |  | 27 | Fig. S1D, excluding the effects of sex and reproductive status on *Mycoplasma* and the effects of sex and age on *Bartonella* | 0 |
|  |  | 28 | Fig. S1D, excluding the effects of sex and age on *Mycoplasma* and the effects of reproductive status and sex on *Bartonella* | 0 |
|  |  | 29 | Fig. S1D, excluding the effects of age and sex on *Bartonella* | 0 |
| Longitudinal | Indirect interactions | 30 | Fig. S1E | 0 |
|  |  | 31 | Fig. S1E, excluding the effect of *Mycoplasma* on *Bartonella* | 2 |
|  |  | 32 | Fig. S1E, excluding the effect of sex on *Bartonella* | 1 |
|  |  | 33 | Fig. S1E, excluding the effects of *Mycoplasma,* sex and age on *Bartonella* | 5 |
|  |  | 34 | Fig. S1E, excluding the effect of age on fleas | 1 |
|  |  | 35 | Fig. S1E, excluding the effect of reproductive status on fleas | 0 |
|  |  | 36 | Fig. S1E, excluding the effects of age and sex on fleas | 2 |
|  |  | 37 | Fig. S1E, excluding the effect of age on ticks | 0 |
|  |  | 38 | Fig. S1E, excluding the effect of reproductive status on ticks | 0 |
|  |  | 39 | Fig. S1E, excluding the effect of *Mycoplasma* on *Bartonella* and the effects of age and sex on fleas, ticks and *Bartonella* | 0 |
|  |  | 40 | Fig. S1E, excluding the effect of sex on fleas, ticks and *Bartonella* | 0 |
|  |  | 41 | Fig. S1E, excluding the effect of reproductive status on fleas, ticks and *Bartonella* | 0 |
|  |  | 42 | Fig. S1E, excluding the effect of *Mycoplasma* on *Bartonella* and the effects of sex and reproductive status on fleas, ticks and *Bartonella* | 0 |
|  |  | 43 | Fig. S1E, excluding the effects of sex and age on fleas, ticks and *Bartonella* | 0 |
|  |  | 44 | Fig. S1E, excluding the effects of sex and reproductive status on fleas, ticks and *Bartonella* | 0 |
|  |  | 45 | Fig. S1E, excluding the effect of sex on fleas and *Bartonella* and the effects of sex, age and *Mycoplasma* on *Bartonella* | 9 |
|  |  | **46** | **Fig. 2, including the effect of reproductive status on *Bartonella* and excluding the effect of age on *Bartonella* and fleas** | **16** |
|  |  | 47 | Fig. S1E, excluding the effects of sex and age on fleas, the effect of sex on ticks and the effects of sex, age and *Mycoplasma* on *Bartonella* | 0 |
|  |  | **48** | **Fig. 2, excluding dashed arrows** | **37** |
|  |  | **49** | **Fig. 2, including the effect of age and excluding the effect of reproductive status on fleas** | **25** |

**Fig. S1. Basic path models used for the construction of the set of competing models (Table S1)**

Models in each set were compared in order to find the best models that predict the community composition and the determinants of the most dominant parasites associated with *G. andersoni* rodents (*S. cleopatrae* fleas, *H. impeltatum* ticks, and *Mycoplasma* and *Bartonella* bacteria) and the temporal changes of this community as determined by the [[cross-sectional (‘CS’; A-D) and](http://en.wikipedia.org/wiki/Cross-sectional_study) longitudinal data](http://en.wikipedia.org/wiki/Cross-sectional_study)sets (‘LONG’; E), respectively. All models include the most important associations revealed in stage 1 (marked by bold in Table 1) but vary in the predicted causal links between variables. Details on the Akaike weights and the full model set derived from these basic models are provided in Table S1.
